# Enhancing Health Equity and Patient Engagement in Diabetes Care: Technology-Aided Continuous Glucose Monitoring Pilot Implementation Project

**DOI:** 10.2196/68324

**Published:** 2025-02-05

**Authors:** Madhur Thakur, Eric W Maurer, Kim Ngan Tran, Anthony Tholkes, Sripriya Rajamani, Roli Dwivedi

**Affiliations:** 1 Institute for Health Informatics Medical School University of Minnesota Minneapolis, MN United States; 2 Community-University Health Care Center Office of Academic Clinical Affairs University of Minnesota Minneapolis, MN United States; 3 Clinical and Translational Science Institute Office of Academic Clinical Affairs University of Minnesota Minneapolis, MN United States; 4 School of Nursing University of Minnesota Minneapolis, MN United States; 5 Department of Family Medicine and Community Health Medical School University of Minnesota Minneapolis, MN United States

**Keywords:** consumer health informatics, patient engagement, diabetes mellitus, DM, glucose monitoring, continuous glucose monitoring, CGM, health equity, health information technology, patient centered care, diabetes, pharmacists, clinicians, nurses, device, patient monitoring, technology-aided, health informatics

## Abstract

Federally Qualified Health Centers (FQHCs) provide service to medically underserved areas and communities, providing care to over 32 million patients annually. The burden of diabetes is increasing, but often, the vulnerable communities served by FQHCs lag in the management of the disease due to limited resources and related social determinants of health. With the increasing adoption of technologies in health care delivery, digital tools for continuous glucose monitoring (CGM) are being used to improve disease management and increase patient engagement. In this viewpoint, we share insights on the implementation of a CGM program at an FQHC, the Community-University Health Care Center (CUHCC) in Minneapolis, Minnesota. Our intent is to improve diabetes management through better monitoring of glucose and to ensure that the CGM program enables our organization’s overarching digital strategy. Given the resource limitations of our population, we provided Libre Pro devices to uninsured patients through grants to improve health care equity. We used an interdisciplinary approach involving pharmacists, nurses, and clinicians and used hemoglobin A1c (HbA1c) levels as a measure of diabetes management. We assessed the CGM program and noted key aspects to guide future implementation and scalability. We recruited 148 participants with a mean age of 54 years; 39.8% (59/148) self-identified their race as non-White, 9.5% (14/148) self-identified their ethnicity as Hispanic or Latino, and one-third (53/148, 35.8%) were uninsured. Participants had diverse language preferences, with Spanish (54/148, 36.5%), English (52/148, 35.1%), Somali (21/148, 14.2%), and other languages (21/148, 14.2%). Their clinical characteristics included an average BMI of 29.91 kg/m2 and a mean baseline HbA1c level of 9.73%. Results indicate that the CGM program reduced HbA1c levels significantly from baseline to first follow-up (P<.001) and second follow-up (P<.001), but no significant difference between the first and second follow-up (P=.94). We share key lessons learned on cultural and language barriers, the digital divide, technical issues, and interoperability needs. These key lessons are generalizable for improving implementation at FQHCs and refining digital strategies for future scalability.

## Introduction

### Growing Burden of Diabetes

Diabetes mellitus is a chronic metabolic, autoimmune, and genetic disease involving elevated levels of blood glucose [1,2]. It poses a significant public health challenge globally as the estimated prevalence of diabetes among people aged 20-70 years was 10.5% in 2021, or approximately 536 million people. It is expected to rise to 12.2% (783.2 million people) by 2045. The burden of diabetes is rising among vulnerable populations too, because they frequently face obstacles to effective diabetes management [3,4]. According to the Health Center Program Uniform Data System by Health Resource and Service Administration (HRSA), the percentage of patients with diabetes has been increasing in the last 5 years [5].

### Digital Technology for Diabetes Management, Patient Engagement, and Health Equity

Current health care processes are increasingly utilizing digital technology to provide innovative solutions for patient care and management [6]. One example is remote patient monitoring (RPM) technologies, such as continuous glucose monitoring (CGM) devices, which are becoming an important tool used in diabetes management [7-9]. The CGM devices provide continuous monitoring of blood glucose levels, thereby offering an all-encompassing picture of glucose fluctuations throughout the day and night [8,10]. In contrast to conventional glucose monitoring methods, which require intermittent finger stick tests, CGM devices use sensors positioned under the skin to measure sugar levels continuously [11-13]. This real-time data help patients and clinicians to make decisions about identifying appropriate drugs for intervention and adjusting drug therapy. The patient can also make changes in lifestyle or dietary choices based on monitoring information. These interventions by clinician and patient can lead to better diabetes management [14-16].

Evidence suggests that an underserved population could benefit from digital technologies like CGM. However, many obstacles still exist in providing service to these communities [17,18]. From the health care provider’s perspective, these challenges include a lack of infrastructure, insufficient staffing, lack of electronic data exchange, and limited patient engagement capacity [19,20]. From a patient’s perspective, inadequate broadband access, language barriers, and lack of digital literacy are some important barriers to accessing digital health [20-22]. The limited literature on RPM and telehealth outcomes among racial minority populations and vulnerable groups indicates that health care disparities still exist and stresses the need for targeted efforts to overcome these barriers [8,23].

### Prior Research

Evidence has emerged that shows that the use of RPM in health care settings helps reduce hemoglobin A_1c_ (HbA_1c_) levels in patients with type 2 diabetes [24-26]. In addition, research also suggests that CGM devices show higher acceptance by patients, help in lowering HbA_1c_ levels, and reduce incidences of hypoglycemic events [27]. A pilot study provided evidence for the feasibility of using CGM devices such as Libre Pro in medically vulnerable and underserved populations at a Federally Qualified Health Center (FQHC). It also showed that this digital technology can be used in resource-constraint organizations like primary care health centers [28]. However, the prescription of CGM devices is low in Black and Hispanic populations in comparison to their White counterparts. At the same time, the rate of diabetes is higher in the Black and Hispanic populations [29-31].

### Population Served and Services at the Community-University Health Care Center

Our health care clinic, the Community-University Health Care Center (CUHCC) was founded in 1966 by 2 University of Minnesota pediatricians and is the first and longest-running Community Health Center in Minnesota [32]. It is an FQHC providing comprehensive primary care services to the medically underserved area/population and is funded by the HRSA [33,34]. The CUHCC, being an FQHC, provides services to everyone regardless of their ability to pay and offers sliding scale fees. This makes sure that care is available to all patients regardless of their insurance status, which plays a role in reducing health care inequities [34,35]. The CUHCC provides medical, dental, mental health, and social services to about 10,000 patients a year across 49,000 visits annually. It operates with approximately 170 full-time equivalent (FTE) staff members, have an operating budget of US $26 million, and supports over 170 learners annually [36]. The CUHCC serves a diverse and underserved population, with 91% of patients having a known income level at or below 200% of the federal poverty guidelines in 2021. Of the patient population, 29% identify as Hispanic and 37% as BIPOC (Black, Indigenous, and People of Color). In 2022, close to half (48%) of the CUHCC’s patients preferred a language other than English for their care. A majority of CUHCC patients are covered by Medicaid/Children’s Health Insurance Program (57%) or uninsured (25%), reinforcing its role as a critical health care safety net for vulnerable populations. The burden of diabetes in our population is higher than the national statistics, per HRSA data [5,37].

### Project Objective

Recognizing these gaps, we implemented a CGM program at our site, the CUHCC. Our objective is to share insights on the implementation and outcome of the CGM program for diabetes management among the CUHCC’s patient population and to enumerate lessons learned for an overarching digital strategy for our organization.

## Methods

### Study Eligibility Criteria and Approach

Patients were eligible for the CGM program if they had established care at the CUHCC, were aged 18 years or older, and had been diagnosed with diabetes. Clinicians and nurse practitioners introduced the option of CGM to eligible patients during routine visits. Patients who agreed to participate in the program were scheduled for enrollment visits with clinical pharmacists.

**Figure 1:**
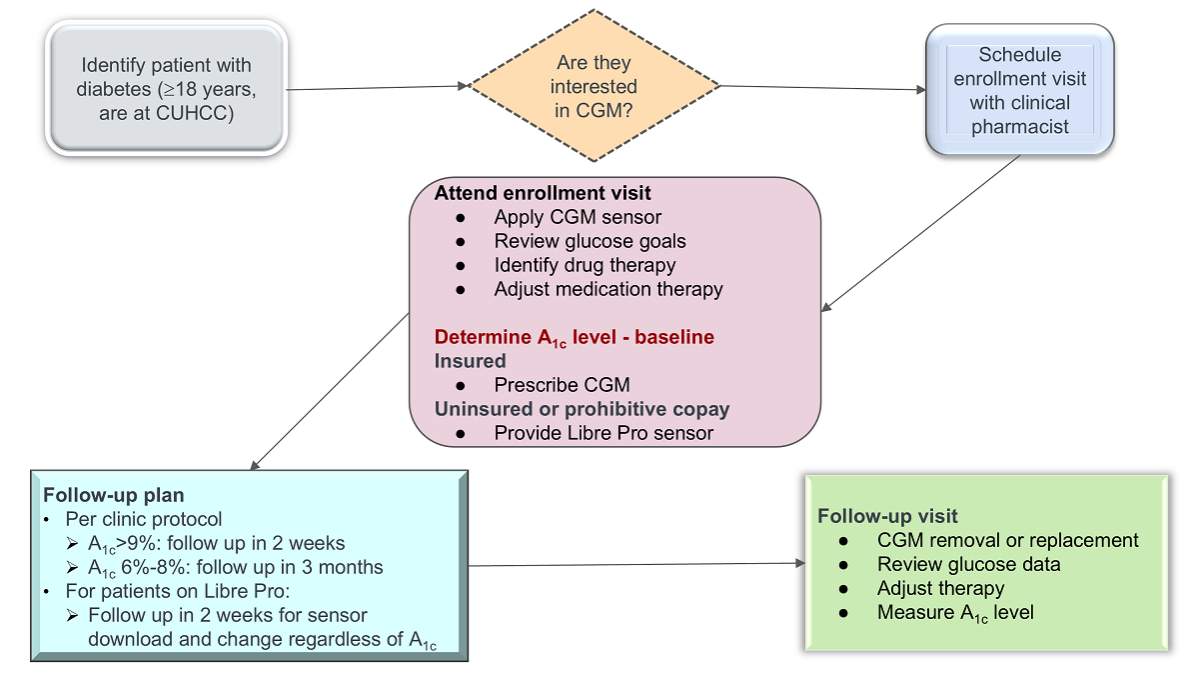
Process for CGM implementation. CGM: continuous glucose monitoring; CUHCC: Community-University Health Care Center.

Our pilot implementation study of CGM was led by a pharmacist team, which consisted of 1.2 FTE clinical pharmacists and 2 FTE pharmacy residents. This interdisciplinary approach with recruitments by clinicians and nurses and follow-up by pharmacy team was chosen based on the evidence that collaborative health care teams are effective in integrating digital health in primary care settings [38,39]. The detailed schema of our approach is depicted in Figure 1.

### CGM Program Protocol and Analysis

The CGM program followed a structured protocol (refer to Figure 1). During enrollment and subsequent visits, pharmacists were responsible for the application and removal of CGM sensors. They also provided patient education and instructions on how to use CGM devices. There was no real-time monitoring of CGM data given the technological barriers, but in follow-up visits, pharmacists downloaded and reviewed the glucose data and adjusted patients’ medications. Follow-up visits were scheduled according to the patient’s HbA_1c_ levels. For patients with HbA_1c_ levels greater than 9%, follow-up visits were recommended every 2 weeks. In contrast, patients with HbA_1c_ levels between 6% and 8% (relatively stable glucose control) were scheduled for follow-up every 3 months. For patients receiving Libre Pro sensors from the clinic, follow-up is required every 2 weeks for sensor download and replacement. The program ensured CGM device accessibility to all eligible patients. For insured patients, a CGM device was prescribed and dispensed through their pharmacy. The CUHCC used Libre Pro CGM sensors donated by the funder to patients who were uninsured or those with unaffordable copays. We defined the outcome measure of interest as the change in levels of HbA_1c_ over time. Baseline HbA_1c_ level is defined as the result closest in time prior to CGM enrollment. Follow-up HbA_1c_ level is defined as the first and second results (about 3 months after the baseline HbA_1c_ level and 6 months after the baseline HbA_1c_ level, respectively) after CGM enrollment.

To assess the effectiveness of the CGM program, a repeated measure ANOVA with Greenhouse-Geisser correction and post hoc pairwise comparisons with Bonferroni correction was performed. These tests are used to determine if there is a statistically significant difference in mean HbA_1c_ level between 3 points: baseline, first follow-up, and second follow-up.

### Lessons Learned

We enumerated the key takeaways from this project with a team-based approach involving key stakeholders in the program including the lead pharmacist and the data analyst. The chief executive officer and the chief innovation & strategy officer, both of whom are advocates for digital technology to address health equity, were an integral part of this collaborative effort.

### Ethical Considerations

This study was a quality improvement project at the CUHCC and did not require institutional review board determination. Participation was voluntary, and patients verbally consented to participate in the CGM program. Program details were shared with participants including data protection, sharing of data from devices, use of individual data for diabetes management, and deidentified data for secondary purposes. One patient opted out of data sharing and their data were removed from this program evaluation. There was no monetary compensation for participation in this project. Patients who were not able to afford the CGM sensor were provided with Libre Pro CGM sensors, which were donated to the CUHCC by Abbott.

## Results

### Demographics and Clinical Characteristics

There were 149 patients who were enrolled in the CGM program at the CUHCC from January 20, 2022, to September 27, 2023. One patient opted out of sharing their medical records and was excluded from the analysis. As shown in Table 1, the patient cohort had a mean age of 54 years, ranging from 19 to 86 years, and consisted of 54.7% (n=81) female participants. The cohort was racially diverse, with one-third (50/148, 33.8%) being Black and African American, 4.1% (6/148) being American Indian or Alaska Native, and 2% (3/148) being Asian. There were 18 (12.2%) patients whose race was unknown, and the rest identified as White (71/148, 48%). In terms of ethnicity, 9.5% (n=14) of the patients identified as Hispanic or Latinx, and ethnicity was not documented for 29.7% (n=44) of patients. Table 1 also shows that the group had a diversity of language preferences, with one-third speaking Spanish (54/148, 36.5%), followed by English (52/148, 35.1%), Somali (21/148, 14.2%), and other languages (21/148, 14.2%). In terms of insurance, approximately one-third (53/148, 35.8%) were uninsured, and the rest (95/148, 64.2%) were insured. The average BMI of the participants was 29.91 (SD 7.66) kg/m^2^, with a range from 18.27 to 56.64 kg/m^2^. The baseline HbA_1c_ levels average 9.73% (SD 2.24), with a range from 5% to 14%. Of the 148 patients in the sample, 65 (43.9%) received Libre Pro CGM sensors, which were provided by the CUHCC.

**Table 1:** Sociodemographic and clinical characteristics of participants (n=148).

Variable		Values, n (%)
**Age group (years)**		
	18-40	22 (14.9)
	41-63	91 (61.5)
	64-86	35 (23.6)
**Sex**		
	Female	81 (54.7)
	Male	67 (45.3)
**Race**		
	White	71 (48.0)
	Black or African American	50 (33.8)
	American Indian or Alaska Native	6 (4.1)
	Asian	3 (2.0)
	Unknown	18 (12.2)
**Ethnicity**		
	Hispanic or Latino	14 (9.5)
	Non-Hispanic or Latino	90 (60.8)
	Unknown	44 (29.7)
**Preferred language**		
	Spanish	54 (36.5)
	English	52 (35.1)
	Somali	21 (14.2)
	Other^a^	21 (14.2)
**Insurance status**		
	Insured	95 (64.2)
	Uninsured	53 (35.8)

^a^Other languages were Central Khmer, Hmong, Korean, Oromo, sign language, and Vietnamese.

### HbA_1c_ Level Outcome

A repeated-measure ANOVA with Greenhouse-Geisser correction was used, as the same metric (HbA_1c_) was measured in participants over time, which enabled the ability to attribute differences related to treatments. This test showed that the difference between the mean HbA_1c_ levels among the 3 points (baseline, first follow-up, and second follow-up) was statistically significant (*F*_1.153,113.38_=38.29; *P*<.001). As presented in Table 2, post hoc pairwise comparisons with Bonferroni correction indicated a statistically significant reduction in HbA_1c_ levels from baseline to the first follow-up (*P*<.001) and from baseline to second follow-up (*P*<.001), but no significant difference between the first and second follow-up (*P*=.94).

**Table 2:** Comparison of follow-up hemoglobin A_1c_ (HbA_1c_) measurements.

Time period	HbA_1c_ measurements			
	Mean difference in HbA_1c_ level (%)	SE	95% CI	*P* value
Baseline to first follow-up	–1.66	0.22	2.20 to –1.13	<.001
Baseline to second follow-up	–1.68	0.26	–2.32 to –1.03	<.001
Between first and second follow-up	–0.01	0.156	–0.39 to 0.37	.94

### Lessons Learned

During the implementation of the CGM program, several key lessons were learned that had implications for the future scalability and sustainability of the program, along with laying the groundwork for an overarching digital strategy for the organization (presented in Table 3).

**Table 3:** Lessons learned from technology-aided patient engagement.

Topic		Lessons learned	Program implications
**Patient perspectives**			
	Cultural and language barriers	Diverse patient population requires tailored communication strategies	Enhance staff training in cultural competence and develop multilingual resources
	Patient education	Importance of comprehensive education on CGM^a^ benefits and use	Develop comprehensive patient education materials in multiple languages and provide ongoing support
	Financial barriers	Half of patients (44%) required financial assistance for CGM devices, and this needs to be addressed to promote health equity	Secure funding or subsidies to ensure equitable access
	Follow-up adherence	Effective follow-up based on HbA_1c_^b^ levels requires active communication	Implement robust patient follow-up systems and reminders
	Social drivers of health	Numerous socioeconomic and contextual factors influence health	Develop RPM^c^ in context of SDoH^d^ for sustainability
**Organizational perspectives**			
	Health equity	Technology offers various tools to improve access but needs to focus on digital equity	Ensure that technology implementations have health equity at the forefront
	Digital divide	Some subsets of the population do not have access to technology or the ability to use it	Need for digital navigators for assistance
	Interdisciplinary collaboration	Pharmacist-led approach proved valuable for diabetes management	Foster interdisciplinary teamwork in program design and implementation
	Patient motivation	Maintaining patient motivation over time was challenging	Use motivational strategies and digital tools to keep patients engaged
	Staff time and effort to set up programs	Recognizing that technology implementations do require time and effort to set up	Gain efficiencies quickly to demonstrate ROI^e^ for these programs
**Technical perspectives**			
	Technical barriers	Some patients had difficulties using digital health tools	Provide more extensive technology training support
	Need for interoperability	Data need to flow seamlessly across devices and settings	Address data entry burden for staff by device data integration
	Workflow integration	Integration of CGM data requires adjusting clinic workflows and appointment structures	Design workflows that include specific times for CGM review during patient visits
	Utility of PROM^f^ data	CGM data need to be integrated into clinical decision-making	Explore solutions and national standards to integrate CGM data in EHRs^g^, along with visuals/trends for providers
	Digital strategy	CGM/RPM enables technology-aided patient engagement	Include these tools as part of an overall digital strategy for the organization

^a^CGM: continuous glucose monitoring.

^b^HbA_1c_: hemoglobin A_1c_.

^c^RPM: remote patient monitoring.

^d^SDoH: social drivers of health.

^e^ROI: return on investment.

^f^PROM: patient-reported outcome measure.

^g^EHR: electronic health record.

## Discussion

### Findings and Implications

Our pilot project was able to successfully recruit 148 participants for the CGM program, along with an enumeration of lessons learned. The reduction of HbA_1c_ levels from baseline to follow-up periods demonstrates the potential and possibility of CGM devices in glycemic control. This suggests that CGM is an effective tool for the management of diabetes, even in resource-constrained environments serving diverse patient populations. Along with statistical significance, these results are clinically significant as achieving this reduction in HbA_1c_ level has the immense benefits of reducing complications from diabetes. Our program evaluation also identified several lessons that include education, financial barriers, follow-up adherence, cultural and language barriers, and context around social drivers of health from a patient’s perspective. In terms of organization, the insights for future implementation are health equity, digital divide, staff time and efforts, and patient motivation.

From the technical side, the barriers include the need for interoperability, workflow integration, and the utility of patient-reported outcome measure data. The result of the CGM program at an FQHC builds on recent literature on RPM and CGM in diabetes. For example, a Digital Health Pilot program for diabetes was implemented at a rural FQHC, which led to improvement in HbA_1c_ levels in the participants [31]. Another pilot study demonstrates a reduction of HbA_1c_ levels and a decrease in hypoglycemic episodes after the implementation of CGM program at an FQHC [33]. These findings have implications for future scalability, sustainability of CGM programs, overall RPM programs, and overarching digital strategy for an organization.

### Strengths and Limitations

An important strength of our pilot project is its focus on a diverse and medically underserved population. This is valuable because there is a scarcity of research focused on these communities. The use of an interdisciplinary approach led by pharmacists, clinicians, nurse practitioners, and nutritionists/dieticians is consistent with the growing evidence of the impact of using collaborative models for disease management. Additionally, our program used broad eligibility criteria, ensuring inclusivity and making certain that patients who meet basic requirements get access to the program.

There are several limitations that need to be addressed. First, the program was implemented at a single site and with a limited number of participants. This may limit the generalizability of the findings to other settings, such as rural FQHCs or other private clinics. Second, this pilot project did not include control groups, which may limit our ability to attribute the changes in HbA_1c_ levels solely to CGM intervention.

### Future Directions

This CGM pilot implementation resulted in an improvement in HbA_1c_ levels in patients with diabetes at an urban FQHC serving a diverse, medically underserved patient population. Our program has expanded to include nurses to make it scalable. Given these positive findings, we are exploring options for the continuation of this program, including ongoing collaboration with Abbott for the CGM sensors and pursuing additional sources for support. Additionally, we are planning a qualitative study with interviews to elicit further details about what worked and what is needed to sustain and scale this program. We advocate for additional studies to be conducted in other FQHCs to determine if this can be replicated and if there are site-specific factors that influence implementation and outcomes. Future research needs to evaluate patient and clinician satisfaction with CGM and other related RPM tools.

### Conclusions

Our pilot experience at the CUHCC indicates that the implementation of digital technologies like the CGM program is feasible and effective in the management of diabetes in a diverse and medically underserved population. The future success of our CGM program will depend on addressing the lessons learned and developing an overarching digital strategy for our organization to promote health equity.

## Abbreviations

BIPOC: Black, Indigenous, and People of Color
CGM: continuous glucose monitoring
CUHCC: Community-University Health Care Center
FQHC: Federally Qualified Health Center
FTE: full-time equivalent
HbA1c: hemoglobin A1c
HRSA: Health Resource and Service Administration
RPM: remote patient monitoring

## Data Availability

The datasets generated and analyzed for this study are not publicly available as the data used are protected with individual identifiers and collected as part of the care delivery process.

## References

[1] Egan AM, Dinneen SF (2019). What is diabetes?. Medicine.

[2] Wang M, Tan Y, Shi Y, Wang X, Liao Z, Wei P (2020). Diabetes and sarcopenic obesity: pathogenesis, diagnosis, and treatments. Front Endocrinol (Lausanne).

[3] Sun H, Saeedi P, Karuranga S, Pinkepank M, Ogurtsova K, Duncan BB, Stein C, Basit A, Chan JC, Mbanya JC, Pavkov ME, Ramachandaran A, Wild SH, James S, Herman WH, Zhang P, Bommer C, Kuo S, Boyko EJ, Magliano DJ (2022). IDF Diabetes Atlas: global, regional and country-level diabetes prevalence estimates for 2021 and projections for 2045. Diabetes Res Clin Pract.

[4] Magliano DJ, Islam RM, Barr ELM, Gregg EW, Pavkov ME, Harding JL, Tabesh M, Koye DN, Shaw JE (2019). Trends in incidence of total or type 2 diabetes: systematic review. BMJ.

[5] Minnesota health center program uniform data system (UDS) data. Health Resources and Services Administration.

[6] Frank SR (2000). Digital health care--the convergence of health care and the internet. J Ambul Care Manage.

[7] Farias FACD, Dagostini CM, Bicca YDA, Falavigna VF, Falavigna A (2020). Remote patient monitoring: a systematic review. Telemed J E Health.

[8] Vrany EA, Hill-Briggs F, Ephraim PL, Myers AK, Garnica P, Fitzpatrick SL (2023). Continuous glucose monitors and virtual care in high-risk, racial and ethnic minority populations: toward promoting health equity. Front Endocrinol (Lausanne).

[9] Hayes CJ, Dawson L, McCoy H, Hernandez M, Andersen J, Ali MM, Bogulski CA, Eswaran H (2023). Utilization of remote patient monitoring within the United States health care system: a scoping review. Telemed J E Health.

[10] Hanson K, Kipnes M, Tran H (2024). Comparison of point accuracy between two widely used continuous glucose monitoring systems. J Diabetes Sci Technol.

[11] Al Hayek AA, Robert AA, Al Dawish MA (2019). Differences of FreeStyle libre flash glucose monitoring system and finger pricks on clinical characteristics and glucose monitoring satisfactions in type 1 diabetes using insulin pump. Clin Med Insights Endocrinol Diabetes.

[12] Kirk JK, Stegner J (2010). Self-monitoring of blood glucose: practical aspects. J Diabetes Sci Technol.

[13] Heinemann L, Stuhr A (2018). Self-measurement of blood glucose and continuous glucose monitoring: is there only one future?. Eur Endocrinol.

[14] Karter AJ, Parker MM, Moffet HH, Gilliam LK, Dlott R (2021). Association of real-time continuous glucose monitoring with glycemic control and acute metabolic events among patients with insulin-treated diabetes. JAMA.

[15] Yamashita H, Kato K, Bando H, Kanazawa S, Tanaka M, Sueki E, Kanagawa H, Kawata T, Kawahito A, Aihara A, Miyashiro H (2020). Relationship of glucose variability and daily lifestyle by continuous glucose monitoring (CGM). Asp Biomed Clin Case Rep.

[16] Taylor PJ, Thompson CH, Luscombe-Marsh ND, Wycherley TP, Wittert G, Brinkworth GD (2019). Efficacy of real-time continuous glucose monitoring to improve effects of a prescriptive lifestyle intervention in type 2 diabetes: a pilot study. Diabetes Ther.

[17] Agarwal S, Simmonds I, Myers AK (2022). The use of diabetes technology to address inequity in health outcomes: limitations and opportunities. Curr Diab Rep.

[18] Ebekozien O, Fantasia K, Farrokhi F, Sabharwal A, Kerr D (2024). Technology and health inequities in diabetes care: how do we widen access to underserved populations and utilize technology to improve outcomes for all. Diabetes Obes Metab.

[19] Franciosi EB, Tan AJ, Kassamali B, Leonard N, Zhou G, Krueger S, Rashighi M, LaChance A (2021). The impact of telehealth implementation on underserved populations and no-show rates by medical specialty during the COVID-19 pandemic. Telemed J E Health.

[20] Chen J, Amaize A, Barath D (2021). Evaluating telehealth adoption and related barriers among hospitals located in rural and urban areas. J Rural Health.

[21] Ramsetty A, Adams C (2020). Impact of the digital divide in the age of COVID-19. J Am Med Inform Assoc.

[22] Alkureishi MA, Choo Z, Rahman A, Ho K, Benning-Shorb J, Lenti G, Velázquez Sánchez I, Zhu M, Shah SD, Lee WW (2021). Digitally disconnected: qualitative study of patient perspectives on the digital divide and potential solutions. JMIR Hum Factors.

[23] Andersen JA, Scoggins D, Michaud T, Wan N, Wen M, Su D (2021). Racial disparities in diabetes management outcomes: evidence from a remote patient monitoring program for type 2 diabetic patients. Telemed J E Health.

[24] Salehi S, Olyaeemanesh A, Mobinizadeh M, Nasli-Esfahani E, Riazi H (2020). Assessment of remote patient monitoring (RPM) systems for patients with type 2 diabetes: a systematic review and meta-analysis. J Diabetes Metab Disord.

[25] Lee PA, Greenfield G, Pappas Y (2018). The impact of telehealth remote patient monitoring on glycemic control in type 2 diabetes: a systematic review and meta-analysis of systematic reviews of randomised controlled trials. BMC Health Serv Res.

[26] Kim KK, McGrath SP, Solorza JL, Lindeman D (2023). The ACTIVATE digital health pilot program for diabetes and hypertension in an underserved and rural community. Appl Clin Inform.

[27] Mian Z, Hermayer KL, Jenkins A (2019). Continuous glucose monitoring: review of an innovation in diabetes management. Am J Med Sci.

[28] Sgroi RG, Gumireddy S, Tan WY, Kaplan R, Milambwe Y, Rosoph LH, Salama DA, Feher CR, Rennert NJ (2023). 1034-P: access to continuous glucose monitoring systems in a primary care clinic for underserved patients with type 2 diabetes–a pilot study. Diabetes.

[29] Wallia A, Agarwal S, Owen AL, Lam EL, Davis KD, Bailey SC, DeLacey SE, Pack AP, Espinoza J, Bright D, Eggleston A, Walter E, O'Brien MJ (2024). Disparities in continuous glucose monitoring among patients receiving care in federally qualified health centers. JAMA Netw Open.

[30] Cheng YJ, Kanaya AM, Araneta MRG, Saydah SH, Kahn HS, Gregg EW, Fujimoto WY, Imperatore G (2019). Prevalence of diabetes by race and ethnicity in the United States, 2011-2016. JAMA.

[31] Ariel-Donges AH, Gordon EL, Dixon BN, Eastman AJ, Bauman V, Ross KM, Perri MG (2020). Rural/urban disparities in access to the national diabetes prevention program. Transl Behav Med.

[32] History of CUHCC. Community-University Health Care Center (CUHCC).

[33] 2023 agency overview. Health Resource and Service Administration (HRSA).

[34] Federally qualified health centers (FQHCs) and the health center program. Rural Health Information Hub.

[35] Chapter 9: sliding fee discount program. Health Resources and Services Administration.

[36] About CUHCC. Community-University Health Care Center (CUHCC).

[37] Health center program uniform data system (UDS) data overview. Health Resources and Services Administration.

[38] Krause-Jüttler G, Weitz J, Bork U (2022). Interdisciplinary collaborations in digital health research: mixed methods case study. JMIR Hum Factors.

[39] Yeager S (2005). Interdisciplinary collaboration: the heart and soul of health care. Crit Care Nurs Clin North Am.

