# Correction: Enhancing Health Equity and Patient Engagement in Diabetes Care: Technology-Aided Continuous Glucose Monitoring Pilot Implementation Project

**DOI:** 10.2196/72689

**Published:** 2025-03-20

**Authors:** Madhur Thakur, Eric W Maurer, Kim Ngan Tran, Anthony Tholkes, Sripriya Rajamani, Roli Dwivedi

**Affiliations:** 1 Institute for Health Informatics Medical School University of Minnesota Minneapolis, MN United States; 2 Community-University Health Care Center Office of Academic Clinical Affairs University of Minnesota Minneapolis, MN United States; 3 Clinical and Translational Science Institute Office of Academic Clinical Affairs University of Minnesota Minneapolis, MN United States; 4 School of Nursing University of Minnesota Minneapolis, MN United States; 5 Department of Family Medicine and Community Health Medical School University of Minnesota Minneapolis, MN United States

In Enhancing Health Equity and Patient Engagement in Diabetes Care: Technology-Aided Continuous Glucose Monitoring Pilot Implementation Project” ([JMIR Diabetes 2025;10:e68324]) the authors noted two errors.

In the Ethical Considerations section, the sentence:

Patients who were not able to afford the CGM sensor were provided with Libre Pro CGM sensors, which were donated to the CUHCC by the Abbott Fund.

Has been revised to:

Patients who were not able to afford the CGM sensor were provided with Libre Pro CGM sensors, which were donated to the CUHCC by Abbott.

In the Acknowledgement section, the sentence:

The authors express their gratitude to Abbott Fund for donating the Libre Pro sensors for participants, as well as the Abbott Fund’s ongoing support of digital health programs at the Community-University Health Care Center (CUHCC).

Has been revised to

The authors express their gratitude to Abbott for donating the Libre Pro sensors for participants, as well as the Abbott Fund’s ongoing support of digital health programs at the Community-University Health Care Center (CUHCC).

The correction will appear in the online version of the paper on the JMIR Publications website on March 20, 2025, together with the publication of this correction notice. Because this was made after submission to PubMed, PubMed Central, and other full-text repositories, the corrected article has also been resubmitted to those repositories.

